# Radiological Society of North America (RSNA) 3D printing Special Interest Group (SIG): guidelines for medical 3D printing and appropriateness for clinical scenarios

**DOI:** 10.1186/s41205-018-0030-y

**Published:** 2018-11-21

**Authors:** Leonid Chepelev, Nicole Wake, Justin Ryan, Waleed Althobaity, Ashish Gupta, Elsa Arribas, Lumarie Santiago, David H Ballard, Kenneth C Wang, William Weadock, Ciprian N Ionita, Dimitrios Mitsouras, Jonathan Morris, Jane Matsumoto, Andy Christensen, Peter Liacouras, Frank J Rybicki, Adnan Sheikh, RSNA Special Interest Group for 3D Printing, Abraham Levitin, Adam C Zoga, Alejandro A Espinoza, Alexander J Chien, Amar B Shah, Ambroise Mathurin Dzogang Temdemno, Amin S Chaoui, Amy E Alexander, Anand V Rao, Anne Garcia, Angel R Colon, Antoine Leimgruber, Antoine M Vanderhofstadt, Asra Khan-Bonenberger, Attilio A Guazzoni, Barbara L McComb, Benjamin E Tubb, Benjamin Johnson, Benjamin M Howe, Berdoudi Rabah, Bernadette M Greenwood, Beth A Ripley, Beth M Kline-Fath, Brent Chanin, Brian A Tweddale, Brian McNamee, Bruce M Barack, Bruce M Shuckett, Bryan Crutchfield, Carina L Butler, Carlin A Ridpath, Carlos I Hernandez Rojas, Carlos Torres, Carolina A Souza, Chen C Hoffmann, Cheryl L Kirby, Ching-Lan Wu, Chris Letrong, Christina Kotsarini, Christine J Kim, Christopher A Swingle, Christopher E Smith, Christopher Wilke, Christopher Yurko, Claudio Silva, Colin M Wilson, Craig S Howard, Damodaran Arul Selvam, Dana A Fuller, Daniel A Crawford, Daniel Davis, Daniel LaRussa, Daniel S Madsen, Daniele Marin, Darshit Thakrar, Dave Nuthals, David Dreizin, David M Hough, David MacCutcheon, Daya Vora, Deborah E Starkey, Denis Samama, Derek L West, Diane M Twickler, Donald S Emerson, Dong Xu, Dorothy J Shum, Eddy D Lucas, Eduardo M Rosa, Edward A Del Grosso, Edward P Quigley, Edward Stefanowicz, Enrique R Escobar, Eric M Baumel, Eric Teil, Erik W Stromeyer, Ernest J Ferris, Fabrizio D’Alessandro, Fadi Toonsi, Faisal M Shah, Fernando A Alvarado, Francesco Potito, Frank S Bonelli, Freddy Drews, Gaetano T Pastena, Gary W Kerber, Gene Kitamura, George Antaki, Georgina A Viyella, Gerard P Farrar, Gloria M Rapoport, Gul Moonis, H Henry Guo, Halemane S Ganesh, Han N Ta, Haraldur Bjarnason, Hemant T Patel, Hongju Son, Hui J Chen, Hyun-Ju Lee, Irini M Youssef, Jack M Drew, Jaime Ribeiro Barbosa, James B Allison, James Shin, Jared V Grice, Jaroslaw Ast, Jayanthi Parthasarathy, Jeffrey A Haithcock, Jeffrey A Sodergren, Jeffrey D Hirsch, Jesus D Buonomo, Joaquim M Farinhas, Joel M Stein, Johannes Goerich, John A Skinner, John G O’Rourke, John Oh, John P Knoedler, Jonathan A Aziza, Jonathan M Ford, Jorge E Salazar, Jose A Barriocanal, Jose A Maldonado, Joseph Johnnie, Joseph M Aulino, Josephine Pressacco, Judy H Song, Juergen Brandt, Julie S Lee, Juling Ong I, Justin Sutherland, Karen K Moeller, Katherine Weimer, Kathleen G Oxner, Kathryn E Pflug, Kelly D Smith, Kelly Oppe, Kenneth A Buckwalter, Kenneth L Sandock, Kent R Thielen, Kevin A Lugo, Kevin J Roche, Kevin L Pope, Keyur Mehta, Kimberly Torluemke, Kirby K Wong, Klaus Kubin, Kranthi K Kolli, Kristi B Oatis, Kwok-chung Lai, Lance E Reinsmith, Lauralyn McDaniel, Leizle E Talangbayan, Leszek J Jaszczak, Ligia Cardona, Lincoln Wong, Liza Nellyta, Louis T Kircos, Luc Lacoursiere, Luca Remonda, Lucas M Sheldon, Luigi Grazioli, Luis A Campos, Luis A Rodriguez Palomares, Mamdouh E Rayan, Marc J Gollub, Margaret O Brown, Mariah N Geritano, Mariam Thomas, Mariano Sturla, Mark A Smith, Mark D Alson, Mark E Sharafinski, Marshall B Hay, Mary Ellen Wickum, Mary Hu, Mary L Christie, Mashael K Alrujaib, Matthew Allen, Mayola C Boykin, Melanie Gillies, Michael D Maloney, Michael Gaisford, Michael L Richardson, Michael T McGuire, Michael T Miller, Michael W Itagaki, Michel Berube, Michel D Dumas, Michelle L Walker, Mohammad Eghtedari, Muge Ozhabes, Nathaniel Reichek, Naveen K Gowda, Nicholas C Fraley, Nicholas G Rhodes, Nopporn Beokhaimook, Pamela A Rowntree, Pascal Fontaine, Patricia A Rhyner, Patrick Chang, Paul E Lizotte, Paulo M Bernardes, Pedro E Diaz, Pen-An Liao, Perla M Salgado, Peter M Van Ooijen, Peter Piechocniski, Philip S Lim, Philipp Brantner, Philippe Grouwels, Phillip D Baker, Prasad S Dalvie, Qurashi M Ali Fadlelseed, R Scott Rader, Rajaram E Reddy, Rami M Shorti, Ramin Javan, Randolph K Otto, Raphael J Alcuri, Rasim C Oz, Richard A Levy, Richard E Barlow, Richard K Brown, Richard Shoenfeld, Rikesh J Makanji, Robert A Posniak, Robert L Falk, Robert M DeWitt, Robert S Redlich, Robyn A Pugash, Roy G Bryan, Salim S Merchant, Sang Joon Park, Sang-Sun Han, Sanjay M Mallya, Sanjay P Prabhu, Sankar P Sinha, Sanket Chauhan, Satinder S Rekhi, Scott H Faro, Scott T Williams, Sepideh Sefidbakht, Sergio A Gonzalez, Seth J Berkowitz, Shannon N Zingula, Shannon R Kirk, Sharon W Gould, Shuai Leng, Sidney D Machefsky, Sofiane Derrouis, Srini Malini, Stephane Khazoom, Stephen E Russek, Steven C Horii, Steven R Parmett, Sumit Pruthi, Summer J Decker, Tan M Nguyen, Terence J O’Loughlin, Terry C Lynch, Timothy L Auran, Todd Goldstein, Todd Pietila, Tone Lindgren, Tracy S Chen, Vartan M Malian, Vicente Gilsanz, Victor A McCoy, Vijay Jayaram, Vinicius V Alves, W Brian Hyslop, Wael M Abdalla, Walter A Carpenter, Wellington Eddy Reynaldo Paez Zumarraga, William D Boswell, William Prows, Xing-Jun Gao, Yeong Shyan Lee, Yiwen Chen, Yoshimi Anzai, Zheng Jin, Adrian A Negreros-Osuna, Andreas Giannopoulos, Andres Vasquez, Boris Kumaev, Carissa M White, Eduardo Hernandez-Rangel, Elias Kikano, Elisa Spoldi, Jessica D Shand Smith, Justin Kerby, Kirk P Langheinz, Luis G Ricardez, Michael Bartellas, Narayana Vamyanmane Dhananjaya Kotebagilu, Sadia R Qamar, Sherazad Islam, Vasanthakumar Venugopal, Vjekoslav Kopacin, Yu-hui Huang

**Affiliations:** 1 Department of Radiology and The Ottawa Hospital Research Institute, University of Ottawa, Ottawa, ON Canada; 2 Center for Advanced Imaging Innovation and Research (CAI2R), Bernard and Irene Schwartz Center for Biomedical Imaging, Department of Radiology, NYU School of Medicine, New York, NY USA; 3 Sackler Institute of Graduate Biomedical Sciences, NYU School of Medicine, New York, NY USA; 4 Rady Children’s Hospital, San Diego, CA USA; 5 Department of Diagnostic Radiology, Division of Diagnostic Imaging, The University of Texas MD Anderson Cancer Center, Houston, TX USA; 6 Mallinckrodt Institute of Radiology, Washington University School of Medicine, Saint Louis, MO USA; 7 Baltimore VA Medical Center, University of Maryland Medical Center, Baltimore, MD USA; 8 Department of Radiology and Frankel Cardiovascular Center, University of Michigan, Ann Arbor, MI USA; 9 Department of Neurosurgery, State University of New York Buffalo, Buffalo, NY USA; 10 Department of Radiology, Mayo Clinic, Rochester, MN USA; 11 3D Medical Applications Center, Walter Reed National Military Medical Center, Washington, DC, USA

**Keywords:** 3D printing, Appropriateness, Guideline, Quality, Radiology, Additive manufacturing, Anatomic model

## Abstract

Medical three-dimensional (3D) printing has expanded dramatically over the past three decades with growth in both facility adoption and the variety of medical applications. Consideration for each step required to create accurate 3D printed models from medical imaging data impacts patient care and management. In this paper, a writing group representing the Radiological Society of North America Special Interest Group on 3D Printing (SIG) provides recommendations that have been vetted and voted on by the SIG active membership. This body of work includes appropriate clinical use of anatomic models 3D printed for diagnostic use in the care of patients with specific medical conditions. The recommendations provide guidance for approaches and tools in medical 3D printing, from image acquisition, segmentation of the desired anatomy intended for 3D printing, creation of a 3D-printable model, and post-processing of 3D printed anatomic models for patient care.

## Background

In 2016, the Radiological Society of North America (RSNA) approved a proposal to create the Special Interest Group on 3D Printing (SIG). This document fulfills two of the original SIG goals: to provide recommendations towards consistent and safe production of 3D printed models derived from medical images, and to describe a set of clinical scenarios for 3D printing is appropriate for the intended use of caring for patients with those medical conditions. This project also fills a previously unmet need for practice parameters/guidelines regarding the clinical service of anatomic modeling (3D Printing) described for proposed new billing codes, including those for the American Medical Association. These practice parameters and recommendations are not intended as comprehensive standards but do reflect several salient aspects of clinical anatomic modeling and appropriateness. The guidelines subcommittee of the SIG will maintain and devote the time and effort necessary to continually develop and update these recommendations. This subcommittee is comprised of volunteer members of the SIG who form the writing group of this document.

In its current state, medical 3D printing [1–576] has been performed for a variety of patients, but without evidence-based appropriateness guidelines. For many body parts, this document includes a comprehensive assessment of appropriateness from the medical literature, supplemented by expert opinion (SIG members) when there is a paucity of peer-review data. After the clinical decision to use 3D printing for patient care, there are many subsequent steps, as reviewed in prior literature [563, 566, 577]. These include image acquisition, image segmentation (demarcation of the desired 3D anatomy), creating 3D-printable file types for each segmented part, printing, and post processing of 3D medical models. This document differs from existing works, including case reports, small and larger studies, and 3D printing review articles in the literature. This is not a review article; instead of reviewing the literature or providing data regarding the clinical utility of medical 3D printing, the RSNA SIG has assembled a group of experts to begin to provide consensus recommendations on the practice of medical modeling and 3D printing, particularly for practice within healthcare facilities. 3D printing of anatomical models within a hospital has recently become recognized as point-of-care manufacturing. These recommendations create a foundational outline to provide practice recommendations for those steps required for medical 3D printing, including image acquisition, segmentation, printing, post-processing, and model verification.

## Methods

### Consensus methodology recommendations

The recommendations regarding medical image acquisition, image data preparation and manipulation, generation of 3D printed models, quality control, communication with referring physicians, preoperative planning using 3D printed models, and considerations regarding materials were discussed and summarized by members of the RSNA Special Interest Group for 3D Printing during several meetings, including on August 31 (Silver Spring, MD) and December 1, 2017 (Chicago, IL) after review of the relevant medical 3D printing literature [1–576] and the local clinical practice of representative members of the Special Interest Group. Relevant recommendations were further exposed to internal online discussion and summarized by a focused taskforce. The final recommendations were reviewed and vetted by all RSNA 3D printing SIG members.

### Appropriateness consensus guideline generation

The Special Interest Group has initiated the quality and safety scholarship to identify those clinical situations for which 3D Printing is considered an appropriate, and not appropriate, representation of the data contained in a medical imaging examination. These documents highlight appropriateness of medical 3D printing for clinical utilization, research, scientific, and informational purposes. This work is loosely modeled after the American College of Radiology Appropriateness Criteria® [553, 554] in that the guidelines committee uses an evidence-based approach at scoring. Consensus among members is used when there is a paucity of evidence.

Each category was led by a separate writing group, composed of a small group of experts in that domain of medical imaging and 3D printing. The SIG Executive committee, led by the Guidelines Chairperson, formed the review panel. Ratings were generated via by a vote of Special Interest Group members at in-person meetings. The results of the ratings follow the following 1–9 format (with 9 being the most appropriate):

- 1–3, red, rarely appropriate: There is a lack of a clear benefit or experience that shows an advantage over usual practice.
- 4–6, yellow, maybe appropriate: There may be times when there is an advantage, but the data is lacking, or the benefits have not been fully defined.
- 7–9, green, usually appropriate: Data and experience shows an advantage to 3D printing as a method to represent and/or extend the value of data contained in the medical imaging examination.

The supporting evidence was obtained through structured PubMed searches, as detailed in the Appendix. In rare circumstances, supporting literature was recommended directly by the members of the committee and was explicitly identified outside of the structured PubMed search results.

A subset of applications of 3D printing, including in congenital heart, vascular, craniomaxillofacial, musculoskeletal, genitounirary, and breast pathologies was selected for detailed review. All final components of this section were vetted and approved by vote of Special Interest Group members at several face-to-face meetings including on August 31 (Silver Spring, MD) and December 1, 2017 (Chicago, IL) as well as via internal posting on the SIG member intranet.

## Results

### Consensus methodology recommendations

#### Medical image acquisition

The most common medical imaging modalities for 3D printing are computed tomography (CT) and magnetic resonance imaging (MRI); however, any 3D imaging dataset including sonography (e.g., echocardiography) may be utilized as input data for segmentation. The international standard format for these imaging files is Digital Imaging and Communications in Medicine (DICOM). At this time, DICOM images are not routinely sent directly to a 3D printer for printing, so medical images are segmented and converted to a file type that is recognized by 3D printers. Common file types include Standard Tessellation Language (STL), OBJ, VRML/WRL, AMF, 3MF, and X3D. Once this functionality is implemented by 3D printing vendors, picture archiving and communication system (PACS) vendors, and at the point of care facility, it will allow 3D files in the form of STLs, for example, to be stored in a patient’s medical record.

##### Spatial resolution and slice thickness

Medical imaging data should have sufficient spatial resolution to accurately represent the anatomy to be modeled. The spatial resolution of an imaging method refers to the smallest resolvable distance between two different objects or two different features of the same object. Low spatial resolution techniques will be unable to differentiate two adjacent structures that are close together and have similar tissue properties. When the intent to produce a 3D model is known prior to a medical imaging procedure, the image acquisition should be tailored so that the anatomy in the intended 3D model can be adequately visualized. The optimal spatial resolution will depend on the anatomy being imaged.

Slice thickness, which influences the spatial resolution and image noise (discussed in the next section), can also be optimized depending on the intended use. In general, this means that the smallest anatomy of interest should be captured on multiple sequential DICOM images of a particular series. For example, if the anatomy of interest measures 3 mm, it would be desirable for this anatomy to be captured on at least 3 sequential image slices; therefore, the slice thickness should be no greater than 1 mm, and preferably smaller. If images are acquired with a large slice thickness, stair-step boundaries may be seen in the 3D model.

For CT, in combination with scan distance, consideration may be given regarding collimation (the thickness of the X-ray beam) and overlap. Typically, the scan distance and collimation are the same; however, if the slice distance is smaller than the collimation, there will be an overlap which may lead to improved results. Cone Beam CT has technical differences with conventional CT, and often results in a lower patient radiation exposure and subsequently less image contrast that typical clinical CT images. Image artifacts and consistency of SNR throughout the scan can also limit studies. For MRI, voxels may be isotropic or rectangular solids and the resolution may be different in the three dimensions. The size of the voxel depends on the matrix size, the field of view (FOV), and the slice thickness.

In some clinical scenarios, there are patients for which suboptimal imaging data is available, but a separate acquisition is contraindicated. If superior spatial resolution is preferred and CT data is required, that benefit should be weighed against the risk of delivering more radiation to the patient.

##### Signal to Noise Ratio (SNR) and Contrast to Noise Ratio (CNR)

The SNR is a metric of image quality. A higher SNR, all else being equal, implies more trustworthy data for 3D printing. The CNR is the relationship of the signal intensity differences (the contrast) between two regions, scaled to noise. High contrast between various organs in the body is an important feature of medical imaging and is necessary to delineate structures for 3D printing. The SNR and CNR of images used for 3D printing should be comparable to, or superior to, those for “3D visualization”, defined as the comprehensive ensemble of manipulation of a volumetric data set for viewing on a 2D surface such as a computer monitor [563].

If the SNR and/or CNR are inconsistent, or suboptimal, the risks of inaccurate segmentation must be weighed against those of rescanning the patient. Regarding high noise data, a judgment call must be made to determine whether the segmentation operator is capable of delineating the data (e.g. in the case of a cone beam CT image series).

In CT, the X-ray tube voltage may also be adjusted to maximize the signal. A lower kV can be used to increase the enhancement of iodine contrast when building vascular models. In addition, the raw data reconstruction parameters selected may affect the appearance of specific anatomical structures. For example, the reconstruction kernel (image filter) impacts both the spatial resolution and image noise, which must be balanced, based on the application. Typically, kernel options range from “sharp” to “smooth.” Sharpening filters increase edge sharpness at a cost of increasing noise while smoothing filters reduce noise content in images by also decrease edge sharpness. For models with fine structures, such as the temporal bone, a sharp kernel is preferred; and for larger, low contrast models, a smooth kernel is more appropriate. CT is considered the imaging modality of choice for bone imaging and is often used to produce 3D anatomical models of hard tissue structures such as bone. In MRI, the SNR may be improved by performing a volume acquisition (at the expense of time), decreasing noise by reducing the bandwidth, altering the echo time or repeat time, increasing the FOV, decreasing the matrix size, or increasing the slice thickness.

##### Image artifact

The sub-volume of the imaging dataset that will be 3D printed is defined in this document as the printing Region of Interest (ROI). All medical images contain artifact, and image processing steps should be taken to minimize artifact. The ROI should be small enough to enable confident segmentation for 3D printing. There are cases for which medical interpretation is possible (see Image interpretation Section), but 3D printing can be limited by the presence of artifact, motion, or other spatial or noise limitations in DICOM images. When this is the case, we recommend that the model be annotated with documentation of those parts of the ROI where segmentation quality may be limited.

##### Image interpretation

Medical images acquired for a clinical indication should be interpreted with the interpretation being incorporated into the patient medical record. The interpretation should include the ROI being considered for printing. Often, interpretation of the ROI incorporates 3D visualization to enable or enhance diagnosis. Examples of 3D visualization include multi-planar reformatting, maximum intensity projections, and volume rendering. Such interpretations are currently billable in the United States under CPT codes 76376 and 76377.

### Image data preparation and manipulation

#### Image segmentation

Image segmentation is necessary to create 3D printable files from medical images. The segmentation process, which subdivides medical images into anatomical regions, typically begins by importing a set of DICOM images into dedicated image post-processing software. Anatomical regions are selected using a combination of automated and semi-automated tools. Once the desired ROI for 3D printing has been selected, data is interpolated and a surface-based 3D model which describes the 3D geometry of that volume is calculated. To date, the most common, widely used, and accepted file format for medical 3D printed objects is the STL file.

STL files are composed of triangular faces, and the number of these faces can affect anatomical accuracy of a model. Each lab should determine the appropriate number of faces/triangles for their medical models to adequately represent anatomy. Operators should be aware of any reduction, smoothing, or further file manipulation or optimization within the segmentation software when creating and exporting the STL file.

The contours of the STL file should be routinely checked against the source medical imaging data; typical segmentation software packages allow the final STL to be re-imported and its contours displayed over the original DICOM images. This option can be used to verify the surface accuracy of an anatomical model STL file. Additional file formats noted above should also meet the same criteria.

#### Segmentation and Computer Aided Design (CAD) software

Medical image processing software is required to generate a file format amenable to 3D printing. The RSNA 3D printing SIG concurs with the FDA that software that has been favorably evaluated by the FDA be used to translate medical images into formats amenable to 3D printing for all aspects of patient care, defined by the SIG as all interactions with healthcare professionals, or patients and their families, related to medical care. The SIG recommends that software used for segmentation is FDA cleared to produce 3D Printed models suitable for diagnostic use, specifically using the FDA definition of diagnostic use and noting that FDA cleared software for 3D printed models will also include machines and materials validated for this intended use. At the time of manuscript submission, the FDA has approved one complete system, consisting of software through the printing process, for medical model production.

#### File storage and descriptors

Files stored within a repository should contain or be linked to a set of corresponding descriptors, including those pertaining to image acquisition and further imaging processing. Descriptors should be supported by standardized terminology from a consensus vocabulary; the SIG acknowledges that this vocabulary represents a current, unmet need. If the descriptors are not within digital files, this information should be otherwise archived.

#### Reference to file manipulation and alteration

Data from medical images undergo alterations in the design of the physical model. These changes have been categorized into Minor and Major alterations [578], with the latter generally representing changes that could impact clinical care. When modifications include major changes, the operator should verify that both the digital file and 3D printed model is labeled/identified appropriately.

### Generation of 3D printed model

#### 3D printing

There are many different 3D printing technologies, each differing in the way that the final 3D printed model is created. When 3D printed models are generated from medical images, the resolution of the 3D printer should be equal to, or superior to that of the clinical images used to segment the model. Similar to the DICOM acquisition stage, it is preferable that printed layers be a multiplier of the smallest geometry of interest. For example, if the smallest anatomical object of interest on the 3D model is 1 mm, this object should be printed on at least 3 layers of the model. Due to the nature of medical models, and the need for sub-millimeter accuracy, a layer thickness of no more than one-third of a millimeter is recommended, and preferably less than or equal to one-eighth of a millimeter. In addition to the layer thickness of the 3D printing hardware, the in-plane (x-y) resolution should be known, with a target of less than one-quarter of a millimeter. The values above are global recommendations may not be applicable in all cases. If a model requires a higher or lower accuracy, these parameters should be modified accordingly.

The medical model should include a patient identifier or an internal unique identification number that can be tracked back to the patient and date of the image acquisition. Labels can be incorporated (3D printed) into the model itself. Labels should be externally attached to the model if size or location does not allow for printed labeling. Printed models are assumed to be of anatomic size (1:1) unless a scaling factor is otherwise noted. Additional identifiers such as model sidedness (left, right) should be noted, as appropriate. Institutional guidelines should be used to verify models are free of protected health information, or models are handled appropriately in accordance to Health Insurance Portability and Accountability Act (HIPAA) guidelines.

#### Post-processing printed models

Post-processing steps should not alter the intended morphology and desired accuracy of the part, but instead should only enhance the utility (including clarity and transparency) and/or durability of the model. It should be noted that finishing may slightly alter the dimensional accuracy of a part, but this variation should be minimal (or within the desired global accuracy of the part) and the benefits (for example: strength and clarity) should outweigh the dimensional change. All support materials and residual manufacturing materials and/or substances should be removed as completely as possible. If all supporting material is not capable of being removed, this should be noted and presented to the requesting provider. Should the model be damaged either during or after post-processing and cleaning, repairs should be performed in a manner that reconstitutes the quality to which the original model adhered. If these repairs are not possible, the model should be reprinted. Any damage should be noted to the provider and the option to reprint should be presented. Cleaning solution concentration and saturation levels should be monitored and maintained in accordance to manufacture recommendations.

#### Model inspection

The model should be inspected by the 3D printing laboratory before clinical use. For cases where the model may be limited by a known image artifact, the model will be noted with any areas of concern. Qualitative and/or quantitative measures to confirm that the 3D printed model matches the desired input data will be taken, including but not limited to expert subjective assessment and objective fitting to the original volume submitted for printing. This can be done on a per part basis, per build basis, or in accordance with an additional internal protocol of the 3D lab. Some examples of qualitative assessments could include comparing the model to a digital representation or printed picture of the model and inspecting the model for printing imperfections or inaccuracies. Some examples of quantitative inspections could include measurements of a test specimen, measurements of the model, or scanning and comparing the model back to the original DICOM data sets.

### The U.S. Food and Drug Administration (FDA)

The U.S. Food and Drug Administration (FDA) ensures the safety and efficacy of personalized devices in the United States of America. 3D Printing falls under the auspices of The Center for Devices and Radiological Health (CDRH). There have been four FDA benchmarks related to 3D printing and medical devices from 2014 to 2018.

First, in October 2014, the FDA held a public workshop entitled “Additive Manufacturing of Medical Devices: An Interactive Discussion on the Technical Considerations of 3D Printing”. Second, the FDA published “Additively Manufactured Medical Products – The FDA Perspective” [579]. Third, in December 2017, the FDA published “Technical Considerations for Additive Manufactured Devices” [580]. This perspective included insights regarding 3D printing data manipulation and hardware for modeling patient-specific anatomy. Fourth, the FDA commented on the publication “Maintaining Safety and Efficacy for 3D Printing in Medicine” [578]. This paper uses a similar, logical 3-step format of these consensus recommendations, and then develops different suggestions for regulatory models that depend on how much, if at all, the anatomical data is modified before 3D printing. On August 31st, 2017, the RSNA SIG and the FDA engaged in a joint meeting to discuss 3D printed anatomic models. The intended output of this meeting is a co-published white paper that will form the next benchmark.

### Quality control program

Due to environmental factors and material properties, model morphology is expected to change over time. As part of a complete quality control program, 3D printers should undergo regular accuracy testing, including test prints, preventative maintenance, and recalibration [581, 582]. Laboratories may develop a process using a phantom to ensure regular quality standards for their printers. If the reference standard is known or assumed, mathematical operations [583] can be applied equally to those volumes in the ROI to determine the overall accuracy of the model, including not only potential manual errors from segmentation, but also generation of the final data set including digital post-processing steps such as smoothing.

### Delivery and discussion with referring physicians

3D printed models represent an advanced form of communication of the data in medical images, and may include the summation of data from multiple sources. Extensive multidisciplinary teaching opportunities for 3D printing have been realized [584–586]. Physicians should have an opportunity to discuss the salient features and intended use of all models. Any concerns about the model or segmentation process, if not discussed previously, should be noted to the provider at the time of delivery. Where possible, annotations detailing critical points of model anatomy should be stored both within the digital record of the model, and physically placed on the 3D printed model. One example is annotation of a subtle fracture that may not otherwise be represented in either or both, the segmented, or the 3D printed model.

### Pre-operative planning

“Pre-operative planning” with 3D printing refers to virtual surgical planning (also called digital templating, digital surgical planning, virtual planning, computerized planning, computer-assisted surgical simulation). This detailed planning of the intervention occurs in the digital space. There are times when the simulation itself is the end product, and the interventionist acquires valuable information regarding patient anatomy and medical devices to be used to increase confidence and knowledge before surgery. For these cases the digital plan is transferred to patient care by way of 3D printed templates, guides, or models. This type of planning usually involves major changes to the digital model while utilizing original patient contours. This necessitates the systematic application of the 3D printing recommendations outlined above to the models used for virtual surgical planning as a minimum requirement.

### Material biocompatibility, cleaning, and sterilization

For anatomical models and surgical guides/templates/jigs potentially entering a surgical field, material biocompatibility, cleaning, and sterilization are vitally important. The details are beyond the scope of this document. However, biocompatibility of materials depends on several factors including base material, the 3D printing process (and any variations), any post-processing techniques, and hospital cleaning and sterilization methods and requirements. Manufacturers should provide cleaning recommendations and specifications for materials which have been formally tested for biocompatibility and sterility, and these specifications should be followed by the facility. Additional internal sterilization policies may exist depending on the hospital.

### Appropriateness of 3D printing (anatomic modeling) for selected clinical scenarios

This section provides evidence-based guidelines, supplemented by expert opinion when there is a paucity of peer-review data, to define and support the use of 3D printing for patients with a variety of conditions, including congenital heart, vascular, craniomaxillofacial, musculoskeletal, genitounirary, and breast pathologies (Table 1).

**Table 1:**
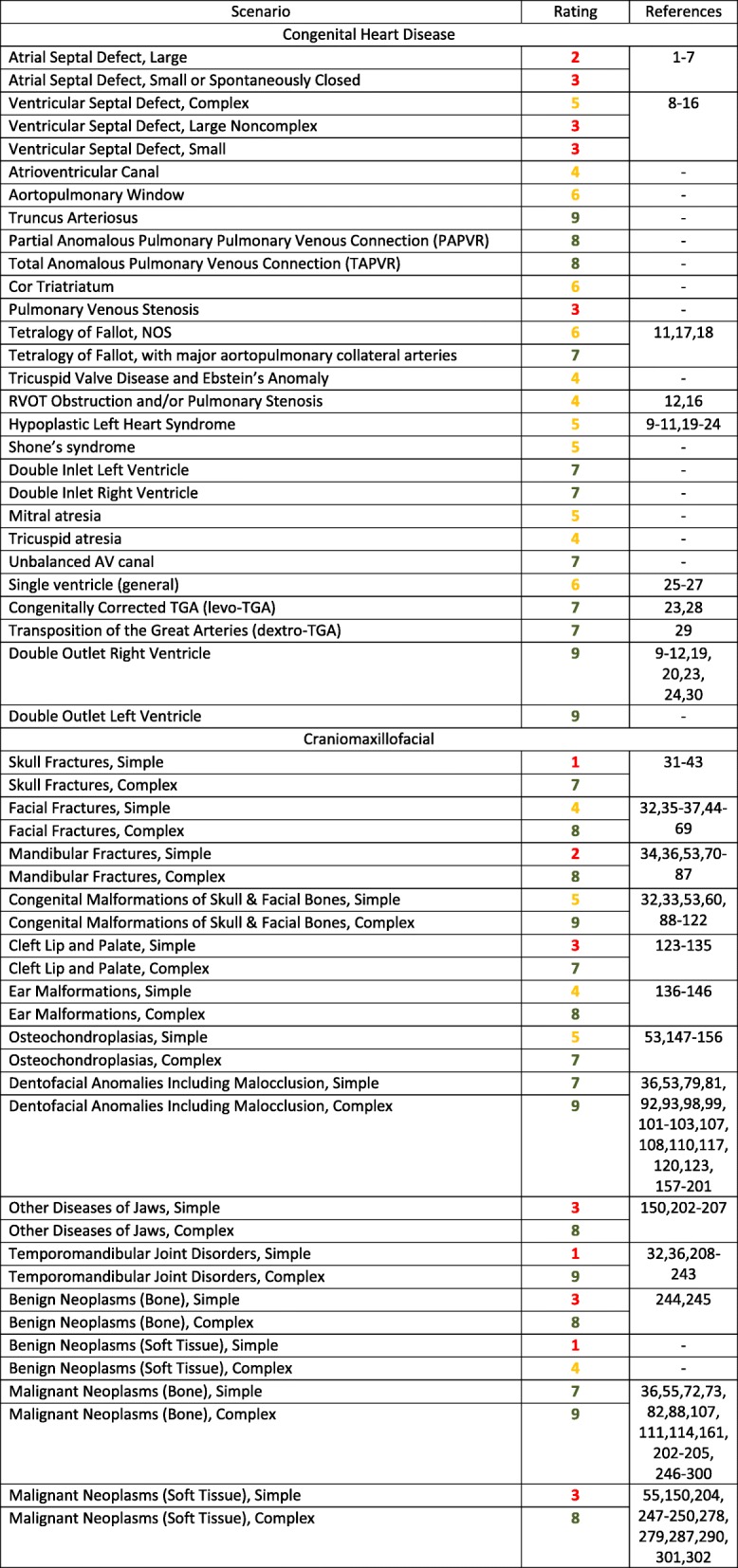

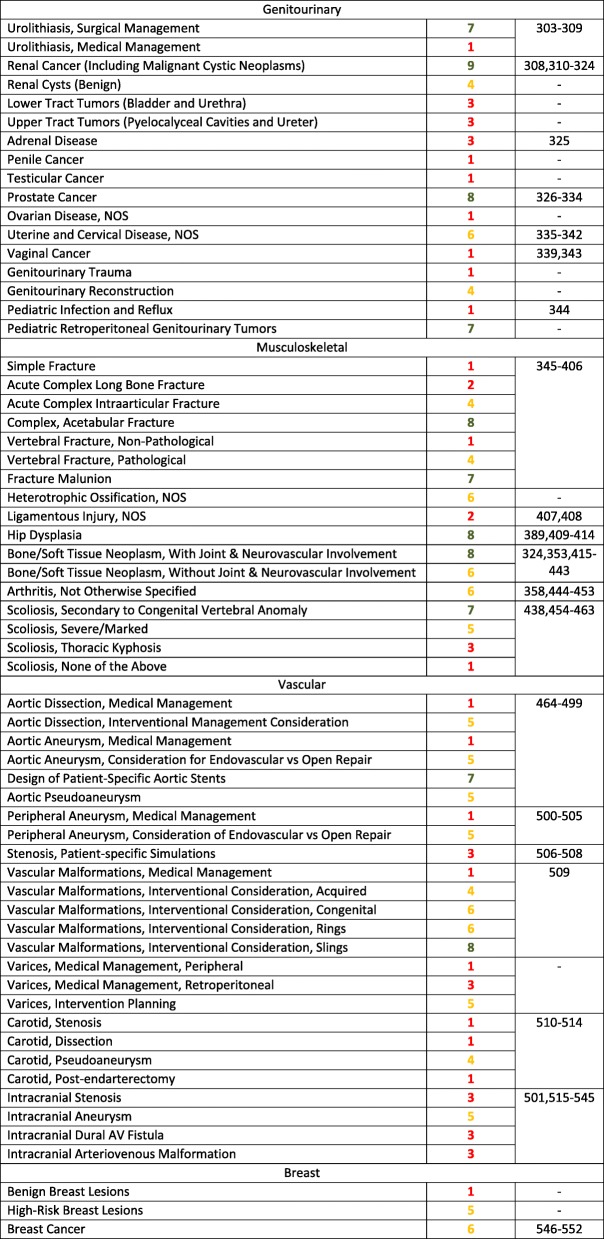
Ratings Summary: Appropriateness Guidelines (scoring system defined in Methods) for patients who present with a variety of medical conditions, and for whom 3D Printing is often considered

## Discussion

Reviews that include the types of 3D printers commonly used in medicine have been published [563, 584]. Regarding image post-processing and software, several tutorials are available for step-by-step training. The following discussion includes the specific descriptions from the SIG writing group for each clinical group of clinical scenarios considered for appropriateness.

### Congenital heart disease

Congenital heart diseases (CHD) are the most common significant birth defects. Substantial literature supports the benefit of 3D printing for patients with congenital heart disease [1–7]. Regarding improved outcomes, precise preoperative understanding of the complex anatomy from a printed model may obviate or shorten lengthy exploration, and therefore operation and cardiopulmonary bypass time can be reduced.

These recommendations utilize and conform to the CHD nomenclature defined by the European Association for Cardio-Thoracic Surgery / Society of Thoracic Surgery (EACTS-STS) version of the International Pediatric and Congenital Cardiac Code (IPCCC), except as where noted otherwise. The clinical scenarios defined by the IPCCC include the following: Septal Defects, Pulmonary Venous Anomalies, Cor Triatriatum, Pulmonary Venous Stenosis, Right Heart Lesions, Left Heart Lesions, Single Ventricle, Transposition of the Great Arteries, DORV, DOLV.

Structured searches were performed using the US National Library of Medicine (PubMed), which enabled the querying and retrieval of appropriate clinical documents supporting the appropriateness of 3D printing-enabled technologies for each specific diagnosis. The search results were reviewed by experts and some references were removed and some were relocated to different categories. As noted above, references outside of the structured searches were added but noted and approved by the writing group. As a general rule, the benefits of 3D printing to define and rehearse an intervention increase with the overall degree of complexity of disease.

### Craniomaxillofacial pathologies

The International Classification of Diseases, Tenth Revision (ICD-10) [555] descriptions and categorization were used to categorize the clinical scenarios for rating craniomaxillofacial conditions. Four major groups were used as the starting point; 1) Craniomaxillofacial Trauma, 2) Congenital Malformations, 3) Acquired/Developmental Deformities and 4) Neoplasms. Further sub-groups were formed underneath the major groupings. Additional clarification for “simple” versus “complex” diagnoses within a particular group was given based on inherent differences in appropriateness ratings between subgroups of patients in these groups. Further language describing each diagnostic grouping helps describe the difference between a simple and a complex case in each subcategory.

Structured searches were performed using the US National Library of Medicine (PubMed), which enabled the querying and retrieval of appropriate clinical documents supporting the appropriateness of 3D printing-enabled technologies for a specific condition. The search results were reviewed by experts and some references were removed because they were not relevant. A small number of references were added because they were found to be relevant, but not appearing using the stated search string. As noted above, these were vetted by the writing group before inclusion. Clinical scenarios that were only dental or only brain have not been included. The authors recognize that these include many important clinical scenarios of for 3D printing, and the goal is to include them in upcoming documents.

Craniomaxillofacial (CMF) conditions for the purposes of this document encompass several different surgical specialties all working in the head and neck area with both pediatric and adult patients. These include oral and maxillofacial surgery, craniofacial surgery, plastic surgery, microvascular surgery, pediatric neurosurgery and otolaryngology. Use of 3D printing-enabled technologies to aid clinical care in the craniomaxillofacial area has been seen from the very advent of 3D printing in the late 1980s [556, 557]. Even before the commercialization of stereolithography there were surgeons, engineers and researchers figuring out more manual ways of converting medical imaging datasets into 3D models [558]. The fit seems clear, CMF surgery has both a functional component, and for most cases an aesthetic component, where the form carries importance along with the functional restoration. In the CMF arena, the use of anatomical models of anatomy is primarily derived from CT and MRI datasets, and also from an increasing volume of cone beam CT datasets. Patient-specific anatomical models are the baseline, but for many of these applications the value of these technologies has been found in either, a) patient-matched implants (for instance temporomandibular joint reconstruction), or b) virtual surgery combined with templates and guides (for instance orthognathic surgery). The scenarios to follow were thought of in this way, with some of them relying heavily on anatomical models alone and some of them relying with increasing importance on the role that digital planning combined with patient-matched implants or templating is playing.

### Genitourinary pathologies

The genitourinary conditions have been organized anatomically, recognizing that common genitourinary interventions are largely based on anatomic considerations. The complication rate after major genitourinary surgeries is reflected in the complexity of the lesion. For example, more complex kidney tumors are associated with longer operative times, warm ischemia times, and greater blood loss [559]. High kidney tumor complexity can also be correlated to the risk of major postoperative complications requiring a secondary intervention [560].

There is a growing body of literature that supports the benefits to patients from 3D printed models. Specifically, 3D printed models may improve comprehension of anatomy and facilitate pre-surgical planning for complex surgical cases, ultimately reducing operation times and improving patient outcomes.

This document describes and provides rating for the clinical scenarios related to 3D printing of genitourinary pathology [561, 562]. Structured searches were performed using the US National Library of Medicine (PubMed), which enabled the querying and retrieval of appropriated clinical documents supporting the appropriateness of 3D printing for a specific diagnosis. As a general rule, the benefits of 3D printing to define and rehearse a genitourinary intervention increases with the overall degree of complexity of the pathology that is represented by the physical model based on a medical imaging study performed in a radiology department.

### Musculoskeletal pathologies

The role of 3D printed models in addressing musculoskeletal pathologies can vary depending on a specific clinical scenario, ranging from aiding in informed consent to use in preoperative planning. Custom fixation plates, surgical osteotomy guides and implants can also be generated from 3D data, allowing for virtual surgery and design of a custom implant that is modeled after the contralateral healthy side. In addition, mock surgeries can be performed on the physical 3D models, allowing for more intuitive problem solving and measurements preoperatively. Such planning alters surgical management for some patients, either by delaying intervention, or by suggesting an alternative approach. Pre-surgical planning can also decrease operating room time and the number of devices and tools that need to be tried and subsequently wasted and/or re-sterilized. In this sense, 3D printing has proven useful for demonstrating musculoskeletal pathology and for planning interventions.

Based on the accumulating evidence, the use of 3D printed models can positively impact numerous metrics associated with musculoskeletal interventions, including patient and physician satisfaction, operative time, blood loss, and the various direct and indirect costs associated with patient-centered decision making regarding management of complex disease. At present, the musculoskeletal pathologies with potential and established 3D printing-enabled management have been broadly categorized into fractures, chronic osseous abnormalities, degenerative disorders, neoplastic pathologies, scoliosis, and miscellaneous specific applications including ligamentous injury and heterotopic ossification.

### Vascular pathologies

3D printing has been shown to be useful for understanding the vascular anatomy, evaluation of hemodynamics, treatment planning (surgical and endovascular) as well as preclinical testing of devices. It has also been used for medical education and procedural training on vascular models [563–566]. There are several clinical scenarios for which 3D printing has been used in the care of patients with vascular disease. Because of the nature of vascular pathology, dissection, aneurysm, and stenosis are often treated with medical management and “watchful waiting”; most patients follow this algorithm, and there is little to no role for 3D printing. However, some patients have a clinical presentation and non-invasive tests that warrant intervention, while others progress from watchful waiting to planned intervention. For many of these patients, 3D printing is appropriate. Of note, coronary 3D printing, and cardiac printing in general falls outside the scope of this document. These clinical scenarios will be discussed in future documents.

Most aortic dissections are treated medically, and for these patients there is no indication for 3D printing. However, 3D printing may be appropriate for planning intervention in complex dissections, and in particular dissections that also have enlargement. Models have been used for planning and simulation of stent deployment [495]. Simulation on models can help in identifying the best projections for angiography, best catheter and wire combinations to navigate the anatomy, in for determining appropriate balloon and stent size as well as position.

Endovascular repair of complex aortic aneurysm involving the origin of branches, extreme angulations, complex neck anatomy, and short landing zones can be quite challenging. Use of 3D printed models can aid understanding of complex anatomy, device selection, and design of prosthesis best suited for patient’s anatomy. These models have shown to be useful in planning procedures and increase operator confidence [491]. 3D printed models have also been used to precisely place fenestrations on stent grafts to treat complex aneurysms [479, 567]. In addition, graft replicas can be tested on patient specific 3D model for suitability before being deployed in patients.

Aortic surgeries, especially in the region of aortic arch and upper abdominal aorta can be quite challenging due to origin of branches, angulation and complex aneurysm neck anatomy. 3D printed models have shown to improve surgeons’ understanding of anatomy and help preoperative planning [485]. Further, 3D printed models can potentially also be used to plan and simulate surgical and endovascular interventions on visceral aneurysms [502, 503]. These models can also be used for designing [486] and testing [568, 576] endovascular devices like catheters, coils, balloons, and stents.

### Breast pathologies

Breast cancer is the most common solid malignancy in women in the United States [570]. The overall lifetime risk of developing breast cancer for women in the United States is 12.4%. Advancements in diagnostic tests and treatments have led to decreasing death rates of 1.8% per year from 2005 to 2014 [570, 571]. Understanding the extent of disease at the time of diagnosis allows appropriate staging and determination of prognosis and survival, in addition to selection of suitable surgical options [572]. Benefits from 3D printed models and its role as an aid to clinical care has been increasingly described in the literature. 3D printed models have the ability of depicting the extent of disease and relationships of sensitive anatomy, thereby possibly reducing operating time, enhancing utilization of new oncoplastic techniques, and improving patient outcomes.

Benign breast diseases are common and include a wide range of entities [573]. The most common of these entities, fibrocystic change, is clinically observed in up to 50% of women and found histologically in 90% of women [573]. Fibroadenomas are the next most common benign breast disease occurring in 15–23% of women [574]. Surgical management of these entities may be needed in cases where cosmesis is altered or when symptom relief is needed. Surgical management may impact developing breast tissue in young women leading to alterations in its proper development [575]. Therefore, careful understanding of the anatomy may minimize the deleterious effects of surgery in benign breast disease.

## Conclusions

3D printing will play an increasingly important role in enabling precision medicine. This document addresses the clinical scenarios where pathology complexity necessitates a transformation of clinical imaging data into a physical model. Adoption of common clinical standards regarding appropriate use, information and material management, and quality control are needed to ensure the greatest possible clinical benefit from3D printing.

This work provides the first comprehensive literature-based guideline document regarding the implementation of 3D printing in clinical practice and details the appropriate scenarios for numerous clinical applications of 3D printing. It is anticipated that this consensus guideline document, created by the members of the RSNA 3D printing special group, will provide the initial reference for method and clinical application standardization. The document and will be substantially expanded and refined, based on expanding clinical applications.

## Appendix

### Search Methodology and Search Results

Structured searches were performed using the US National Library of Medicine (PubMed), which enabled the querying and retrieval of appropriated clinical documents regarding the appropriateness of 3D printing in each of the scenarios.

Congenital Heart Disease (Retrieved August 2017)

**Atrial Septal Defect (ASD)**: Large; small or spontaneously closed

PubMed Search: ((3D printing) AND (ASD)) OR ((rapid prototyping) AND (ASD)) OR ((3D printing) AND (atrial septal defect)) OR ((rapid prototyping) AND (atrial septal defect))

Results: [1–7]

**Ventricular Septal Defect (VSD)**: complex; large (noncomplex); small

PubMed Search: ((3D printing) AND (VSD)) OR ((rapid prototyping) AND (VSD)) OR ((3D printing) AND (ventricular septal defect)) OR ((rapid prototyping) AND (ventricular septal defect))

Results: [8–16]

**Atrioventricular Canal (AV Canal)**

PubMed Search: ((3D printing) AND (AVSD)) OR ((rapid prototyping) AND (AVSD)) OR ((3D printing) AND (atrioventricular septal defect)) OR ((rapid prototyping) AND (atrioventricular septal defect)) OR ((3D printing) AND (AV Canal)) OR ((rapid prototyping) AND (AV Canal)) OR ((3D printing) AND (atrioventricular canal)) OR ((rapid prototyping) AND (atrioventricular canal))

Results: None

**Aortopulmonary window (AP Window)**

PubMed Search: ((3D printing) AND (AP window)) OR ((rapid prototyping) AND (AP Window)) OR ((3D printing) AND (aortopulmonary window)) OR ((rapid prototyping) AND (aortopulmonary window))

Results: None

**Truncus Arteriosus**

PubMed search: ((3D printing) AND truncus) OR ((rapid prototyping) AND truncus)

Results: None

**Partial Anomalous Pulmonary Venous Connection (PAPVR)**

PubMed search: ((3D printing) AND (Anomalous pulmonary)) OR ((rapid prototyping) AND (Anomalous pulmonary)) OR ((3D printing) AND TAPVR) OR ((rapid prototyping) AND TAPVR) OR ((3D printing) AND PAPVR) OR ((rapid prototyping) AND PAPVR)

Results: None

**Total Anomalous Pulmonary Venous Connection (TAPVR)**

PubMed search: ((3D printing) AND (Anomalous pulmonary)) OR ((rapid prototyping) AND (Anomalous pulmonary)) OR ((3D printing) AND TAPVR) OR ((rapid prototyping) AND TAPVR) OR ((3D printing) AND PAPVR) OR ((rapid prototyping) AND PAPVR)

Results: None

**Cor Triatriatum**

PubMed search: ((3D printing) AND (Cor Triatriatum)) OR ((rapid prototyping) AND (Cor Triatriatum))

Results: None

**Pulmonary Venous Stenosis**

PubMed search: ((3D printing) AND (pulmonary venous stenosis)) OR ((rapid prototyping) AND ((pulmonary venous stenosis)) OR ((3D printing) AND (pulmonary vein stenosis)) OR ((rapid prototyping) AND ((pulmonary vein stenosis))

Results: None

**Tetralogy of Fallot (TOF): NOS; accompanied with major aortopulmonary collateral arteries**

PubMed search: ((3D printing) AND TOF) OR ((rapid prototyping) AND TOF) OR ((3D printing) AND tetralogy) OR ((rapid prototyping) AND tetralogy)

Results: [11, 17] Outside of Search, suggested by SIG Members: [18]

**Tricuspid Valve Disease and Ebstein’s Anomaly**

PubMed search: ((3D printing) AND ebstein) OR ((rapid prototyping) AND ebstein) OR ((3D printing) AND ebsteins) OR ((rapid prototyping) AND ebsteins) OR ((3D printing) AND ebstein’s) OR ((rapid prototyping) AND ebstein’s) OR ((3D printing) AND (tricuspid valve disease)) OR ((rapid prototyping) AND (tricuspid valve disease))

Results: None

**RVOT Obstruction and/or Pulmonary Stenosis**

PubMed search: ((3D printing) AND (RVOT obstruction)) OR ((rapid prototyping) AND (RVOT obstruction)) OR ((3D printing) AND (pulmonary stenosis)) OR ((rapid prototyping) AND (pulmonary stenosis))

Results: [12, 16]

**Hypoplastic Left Heart Syndrome**

PubMed search: ((3D printing) AND HLHS) OR ((rapid prototyping) AND HLHS) OR ((3D printing) AND (hypoplastic left)) OR ((rapid prototyping) AND (hypoplastic left))

Results: [9–11, 19–24]

**Shone’s Syndrome**

PubMed search: ((3D printing) AND shones) OR ((rapid prototyping) AND shones) OR ((3D printing) AND shone’s) OR ((rapid prototyping) AND shone’s)

Results: None

**Double Inlet Left Ventricle**

PubMed search: ((3D printing) AND DILV) OR ((rapid prototyping) AND DILV) OR ((3D printing) AND (double inlet left)) OR ((rapid prototyping) AND (double inlet left))

Results: None

**Double Inlet Right Ventricle**

PubMed search: ((3D printing) AND DIRV) OR ((rapid prototyping) AND DIRV) OR ((3D printing) AND (double inlet right)) OR ((rapid prototyping) AND (double inlet right))

Results: None

**Mitral atresia**

PubMed search: ((3D printing) AND (mitral atresia)) OR ((rapid prototyping) AND (mitral atresia))

Results: None

**Tricuspid atresia**

PubMed search: ((3D printing) AND (tricuspid atresia)) OR ((rapid prototyping) AND (tricuspid atresia))

Results: None

**Unbalanced AV canal**

PubMed search: ((3D printing) AND unbalanced) OR ((rapid prototyping) AND unbalanced)

Results: None

**Single ventricle (general)**

PubMed search: ((3D printing) AND SV) OR ((rapid prototyping) AND SV) OR ((3D printing) AND (single ventricle)) OR ((rapid prototyping) AND (single ventricle))

Results: [25–27]

**Congenitally Corrected TGA (levo-TGA)**

PubMed search: ((3D printing) AND (L-TGA)) OR ((rapid prototyping) AND (L-TGA)) OR ((3D printing) AND LTGA) OR ((rapid prototyping) AND LTGA) OR ((3D printing) AND (levo-transposition)) OR ((rapid prototyping) AND (levo-transposition)) OR ((3D printing) AND (l-transposition)) OR ((rapid prototyping) AND (l-transposition)) OR ((3D printing) AND CCTGA) OR ((rapid prototyping) AND CCTGA) OR ((3D printing) AND (CC-TGA)) OR ((rapid prototyping) AND (CC-TGA)) OR ((3D printing) AND (congenitally corrected transposition)) OR ((rapid prototyping) AND (congenitally corrected transposition)) OR ((3D printing) AND (congenitally corrected transposition)) OR ((rapid prototyping) AND (congenitally corrected transposition)) OR ((3D printing) AND (CC-transposition)) OR ((rapid prototyping) AND (CC-transposition))

Results: [23, 28]

**Transposition of the Great Arteries (dextro-TGA)**

PubMed search: ((3D printing) AND (D-TGA)) OR ((rapid prototyping) AND (D-TGA)) OR ((3D printing) AND DTGA) OR ((rapid prototyping) AND DTGA) OR ((3D printing) AND (dextro-transposition)) OR ((rapid prototyping) AND (dextro-transposition)) OR ((3D printing) AND (d-transposition)) OR ((rapid prototyping) AND (d-transposition))

Results: [29]

**Double Outlet Right Ventricle**

PubMed search: ((3D printing) AND DORV) OR ((rapid prototyping) AND DORV) OR ((3D printing) AND (double outlet)) OR ((rapid prototyping) AND (double outlet))

Results: [9–12, 19, 20, 23, 24, 30]

**Double Outlet Left Ventricle**

PubMed search: ((3D printing) AND DOLV) OR ((rapid prototyping) AND DOLV) OR ((3D printing) AND (double outlet left)) OR ((rapid prototyping) AND (double outlet left))

Results: None

Craniomaxillofacial (Retrieved August 2017)

**Skull Fractures:** Fractures of the cranium include the frontal bone, frontal sinus, parietal, sphenoid, temporal, occipital and mastoid bone. Simple fractures would be non-displaced and may not need surgery. Complex fractures may be comminuted and most likely require surgery early for decompression and/or later for cranial reconstruction. Any violation of the dura or brain requires immediate surgery. ICD-10: S02.0 Fracture of Vault of Skull, S02.1 Fracture of Base of Skull

PubMed Search: ((3D Printing) AND (Skull Fracture)) OR ((Rapid Prototyping) AND (Skull Fracture))

Results: [31–43]

**Facial Fractures**: Description: Facial fractures include fractures of the maxilla, zygoma, nasal bones, and frontal bone in addition to the orbit, which is composed of the orbital surface of the maxillary bone, lamina papyracea of the ethmoid bone, lacrimal bone, greater and lesser wings of the sphenoid bone, orbital process of the zygomatic bone, the orbital process of the palatine bone, and the pars orbitalis of the frontal bone. Non-displaced fractures often heal uneventfully and may be managed non-surgically. Displaced fractures---either linear or comminuted---generally require operative repair to avoid functional or esthetic complications. High energy injuries, such as those seen with unrestrained motor vehicle collisions or gunshot wounds, often result in greater three-dimensional disruption and displacement than low energy injuries, which are often the result of assaults and ground level falls. Patient-matched implants may be required for reconstruction of complex injuries, particularly those involving the orbit and zygoma. ICD-10: S02.2 Fracture of Nasal Bones, S02.3 Fracture of Orbital Floor, S02.4 Fracture of Malar, Maxillary and Zygoma Bones

PubMed Search: ((3D Printing) AND (Facial Fracture)) OR ((Rapid Prototyping) AND (Facial Fracture))

Results: [31–43]

**Mandible Fractures**: Mandible fractures include fractures to the condyle, ramus, coronoid process, mandibular angle, body of the mandible or symphysis. Linear fractures are likely to be less three-dimensional as compared to comminuted fractures, which are often significantly displaced. Depending on the complexity of the fracture, the degree of dislocation and the location, open reduction and internal fixation with titanium plates and screws may be required. Secondary reconstruction of complex post-traumatic deformity may also require patient-matched implants. ICD-10: S02.6 Fracture of Mandible (body), S02.61 Fracture of Condylar Process of Mandible, S02.62 Fracture of Subcondylar Process of Mandible, S02.63 Fracture of Coronoid Process of Mandible, S02.64 Fracture of Ramus of Mandible, S02.65 Fracture of Angle of Mandible, S02.66 Fracture of Symphysis of Mandible

PubMed Search: ((3D Printing) AND (Mandible Fracture)) OR ((Rapid Prototyping) AND (Mandible Fracture))

Results: [32, 35–37, 44–69]

**Congenital Malformations of Skull & Facial Bones**: Syndromal or non-syndromal cases characterized mainly by anomalies which may vary from mild to severe and may present with asymmetric involvement of the skull and facial bones. In simple craniosynostosis one or more sutures may be involved. In complex or syndromal craniosynostosis patients may be programmed genetically to grow abnormally and require repeated surgical operations until growth is complete including cranial vault, facial-orbital advancement including maxillae and mandible surgery after eruption of the secondary dentition. Congenital birth defects characterized by incomplete development or absence of face structures, usually affecting one side of the face. Simple cases would include those such as positional plagiocephaly which most likely involve non-surgical treatment. Complex cases for facial reconstruction (for craniofacial macrosomia or hypertelorism, for instance) or total vault reconstruction in an infant require extensive planning and surgical care. Secondary reconstruction in the growing child or adult may require continued surgical care as the skeleton develops further before reaching skeletal maturity. ICD-10: Q75.0 Craniosynostosis, Q75.1 Craniofacial Dysostosis, Q75.2 Hypertelorism, Q75.3 Macrocephaly, Q75.4 Mandibulofacial Dysostosis, Q75.5 Oculomandibular Dysostosis, Q67.0 Congenital Facial Asymmetry, Q67.3 Plagiocephaly

PubMed Search: ((3D Printing) AND (craniosynostosis)) OR ((Rapid Prototyping) AND (craniosynostosis)) OR ((3D Printing) AND (Hypertelorism)) OR ((Rapid Prototyping) AND (Hypertelorism)) OR ((3D Printing) AND (Plagiocephaly)) OR ((Rapid Prototyping) AND (Plagiocephaly)) OR ((3D Printing) AND (Facial Asymmetry)) OR ((Rapid Prototyping) AND (Facial Asymmetry))

Results: [32, 33, 53, 60, 88–122]

**Cleft Lip & Palate**: Cleft lip and cleft palate are birth defects that affect the upper lip, nose, alveolus, soft and or hard palate. The problem can range from a small notch in the lip (simple) to a unilateral or bilateral complete or incomplete involvement of lip, alveolar bone, soft and hard palate with displacement of the palatal muscles. The functional problems associated with cleft lip and/or palate include problems with eating, speech, and eustachian tube malfunction and middle ear effusion requiring grommet tube insertion into the ear drum. Growth may be abnormal requiring jaw surgery. Surgery involves alveolar bone grafting, lip and nose repair, palate repair including palatal muscle repair and closure of the palatal cleft. Later secondary surgery may be necessary. ICD-10: Q35.1 Cleft Hard Palate, Q35.3 Cleft Soft Palate, Q35.5 Cleft Hard Palate and Cleft Soft Palate, Q36.0 Cleft Lip, Bilateral, Q36.1 Cleft Lip, Median, Q36.9 Cleft Lip, Unilateral

PubMed Search: ((3D Printing) AND (Cleft Palate)) OR ((Rapid Prototyping) AND (Cleft Palate)) OR ((3D Printing) AND (Cleft Lip)) OR ((Rapid Prototyping) AND (Cleft Lip))

Results: [123–135]

**Ear Malformations**: Malformations of the ear can include missing portions of the ear, misshapen portions of the ear, malpositioned ears, large ears (macrotia) or small/missing ears (microtia). Simple cases may require surgical excision of extra tissue or a procedure to restrict prominence. Complex cases are typically very complex surgical cases and may require total auricular reconstruction with autogenous tissues. ICD-10: Q17.1 Macrotia, Q17.2 Microtia

PubMed Search: ((3D Printing) AND (Microtia)) OR ((Rapid Prototyping) AND (Microtia)) OR ((3D Printing) AND (Macrotia)) OR ((Rapid Prototyping) AND (Macrotia))

Results: [136–146]

**Osteochondroplasias**: Osteogenesis imperfecta (OI) is a genetic disorder in which bones break easily. Fibrous Dysplasia of the craniomaxillofacial skeleton may result in benign overgrowth of tissue which is fibrous and lacking calcium. Fibrous dysplasia may impact skeletal appearance but the complex cases begin to compromise other vital structures/organs such as the optic nerve and the brain. Simple cases of OI may include bony fractures which will be handled such as in the Trauma (Group A, II or III classification). Complex fibrous dysplasia cases can be very difficult surgically and require three-dimensional removal/sculpting of the mass while paying close attention to close-by vital structures. Surgical replacement of tumor resection can be very complex. ICD-10: Q78.0 Osteogenesis Imperfecta, Q78.1 Polyostotic Fibrous Dysplasia

PubMed Search: ((3D Printing) AND (Osteogenesis Imperfecta)) OR ((Rapid Prototyping) AND (Osteogenesis Imperfecta)) OR ((3D Printing) AND (Fibrous Dysplasia)) OR ((Rapid Prototyping) AND (Fibrous Dysplasia))

Results: [53, 147–156]

**Dentofacial Anomalies Including Malocclusion**: Dentofacial anomalies can include over or undergrowth of either the upper jaw (maxilla) or the lower jaw (mandible). Malocclusion happens when the teeth are not approximating in a way that allows for normal function and can be any combination of one jaw being smaller, larger or asymmetric as compared to the other. Simple cases may involve surgical repositioning of only one of the jaws and typically symmetrical movements. Complex cases typically involve bimaxillary surgery with highly complex movements of both jaws based upon the clinical and radiographic examination. ICD-10: M26.01 Maxillary hyperplasia, M26.02 Maxillary hypoplasia, M26.03 Mandibular hyperplasia, M26.04 Mandibular hypoplasia, M26.05 Macrogenia, M26.06 Microgenia, M26.07 Excessive tuberosity of jaw, M26.1 Anomalies of Jaw-Cranial Base Relationship, M26.11 Maxillary asymmetry, M26.211 Malocclusion Angle Class I, M26.212 Malocclusion Angle Class II, M26.213 Malocclusion Angle Class III, M26.22 Open Occlusal Relationship, M26.220 Open Anterior Occlusal Relationship, M26.221 Open Posterior Occlusal Relationship

PubMed Search: ((3D Printing) AND (Orthognathic Surgery)) OR ((Rapid Prototyping) AND (Orthognathic Surgery)) OR ((3D Printing) AND (Maxillary Hyperplasia)) OR ((Rapid Prototyping) AND (Maxillary Hyperplasia)) OR ((3D Printing) AND (Maxillary Asymmetry)) OR ((Rapid Prototyping) AND (Maxillary Asymmetry)) OR ((3D Printing) AND (Malocclusion)) OR ((Rapid Prototyping) AND (Malocclusion)) OR ((3D Printing) AND (Anterior Open Bite)) OR ((Rapid Prototyping) AND (Anterior Open Bite)) OR ((3D Printing) AND (Posterior Open Bite)) OR ((Rapid Prototyping) AND (posterior open bite)) OR ((3D Printing) AND (virtual surgical planning)) OR ((Rapid Prototyping) AND (virtual surgical planning))

Results: [36, 53, 79, 81, 92, 93, 98, 99, 101–103, 107, 108, 110, 117, 120, 123, 157–201]

**Other Diseases of Jaws**: Other diseases of jaws include inflammatory, infectious, vascular or iatrogenic processes in which bone is remodeled or eroded, such as osteoradionecrosis and osteomyelitis. Bony lesions such as giant cell lesions and benign cysts may require partial resection of the jaw. Uncomplicated cases may require simple excision. Complicated and destructive lesions may involve segmental resection of the mandible or maxilla and reconstruction, typically with autogenous tissues supported by rigid fixation. Secondary reconstruction with patient-matched implants or further free flaps may be required for complex cases. ICD-10: M27.1 Giant Cell Granuloma, Central, M27.2 Inflammatory Conditions of Jaws (osteoradionecrosis, osteomyelitis, others), M27.3 Alveolitis of Jaws

PubMed Search: ((3D Printing) AND (Giant Cell Granuloma Jaw)) OR ((Rapid Prototyping) AND (Giant Cell Granuloma Jaw)) OR ((3D Printing) AND (Osteoradionecrosis)) OR ((Rapid Prototyping) AND (Osteoradionecrosis)) OR ((3D Printing) AND (Osteomyelitis)) OR ((Rapid Prototyping) AND (Osteomyelitis)) OR ((3D Printing) AND (Alveolitis)) OR ((Rapid Prototyping) AND (Alveolitis))

Results: [150, 202–207]

**Temporomandibular Joint Disorders**: Temporomandibular joint disorders relate to a variety of conditions affecting the anatomic and functional characteristics of the temporomandibular joint. Factors contributing to the complexity of temporomandibular diseases are its relation to dentition and mastication and the symptomatic effects in other areas which account for referred pain to the joint. Common diseases are developmental abnormalities, trauma, subluxation, luxation, arthritis, and neoplasia. Simple cases may not need surgical intervention or may require arthroscopy. Cases that involve loss or gain of vertical dimension in the condyle and result in loss of jaw function (malocclusion or range of motion) may require joint total joint replacement and will often rely on patient-matched implants for reconstruction of the joint(s). Complex conditions such as ankylosis of the joint require careful surgical intervention to avoid surrounding vital structures such as nerves and vasculature. ICD-10: M26.601 Right temporomandibular joint disorder, unspecified, M26.602 Left temporomandibular joint disorder, unspecified, M26.603 Bilateral temporomandibular joint disorder, unspecified

PubMed Search: ((3D Printing) AND (Temporomandibular Joint)) OR ((Rapid Prototyping) AND (Temporomandibular Joint)) OR ((Stereolithography) AND (Temporomandibular Joint)) OR ((CAD-CAM) and (Temporomandibular Joint))

Results: [32, 36, 208–243]

**Benign Neoplasms (Bone)**: Bony benign neoplasms of the craniomaxillofacial area may involve the skull, maxilla, orbit, sinuses and mandible. These can range from simple cases where excision of a mass may be required to very complex cases requiring three-dimensional surgical excision and reconstruction. Reconstruction for complex cases may require autogenous tissue or a free flap and may at times also require patient-matched implants or fixation plates. ICD-10: D16.4 Benign neoplasm of bones of skull and face, D16.5 Benign neoplasm of lower jaw bone

PubMed Search: ((3D Printing) AND (Benign Facial Neoplasm)) OR ((Rapid Prototyping) AND (Benign Facial Neoplasm)) OR ((3D Printing) AND (Benign Jaw Neoplasm)) OR ((Rapid Prototyping) AND (Benign Jaw Neoplasm))

Results: [244, 245]

**Benign Neoplasms (Soft Tissue)**: Benign soft tissue neoplasms of the craniomaxillofacial area include lesions of parotid or salivary glands, the lip, the floor of mouth, other parts of the mouth, tonsil, oropharynx, nasopharynx and hypopharynx. Other cutaneous lesions include neurofibromas, gliomas, dermoids, hemangiomas, lymphangiomas and many other rarer tumors. Diagnosis and excision is indicated in all these lesions when possible. Complex cases can include resection and reconstruction of soft tissue and hard tissue concomitantly. Reconstruction for complex cases may require autogenous tissue or a free flap and may at times also require patient-matched implants or rigid fixation. ICD-10: D11.0 Benign neoplasm of parotid gland, D11.7 Benign neoplasm of other major salivary glands, D10.0 Benign neoplasm of lip, D10.1 Benign neoplasm of tongue, D10.2 Benign neoplasm of floor of mouth, D10.3 Benign neoplasm of other and unspecified parts of mouth, D10.4 Benign neoplasm of tonsil, D10.5 Benign neoplasm of other parts of oropharynx, D10.6 Benign neoplasm of nasopharynx, D10.7 Benign neoplasm of hypopharynx, D10.9 Benign neoplasm of pharynx, unspecified

PubMed Search: ((3D Printing) AND (Benign Parotid Neoplasm)) OR ((Rapid Prototyping) AND (Benign Parotid Neoplasm)) OR ((3D Printing) AND (Benign Salivary Gland Neoplasm)) OR ((Rapid Prototyping) AND (Benign Salivary Gland Neoplasm)) OR ((3D Printing) AND (Benign Neoplasm Tonsil)) OR ((Rapid Prototyping) AND (Benign Neoplasm Tonsil)) OR ((3D Printing) AND (Benign Neoplasm Oropharynx)) OR ((Rapid Prototyping) AND (Benign Neoplasm Oropharynx)) OR ((3D Printing) AND (Benign Neoplasm Nasopharynx)) OR ((Rapid Prototyping) AND (Benign Neoplasm Nasopharynx)) OR ((3D Printing) AND (Benign Neoplasm Hypopharynx)) OR ((Rapid Prototyping) AND (Benign Neoplasm Hypopharynx))

Results: No Relevant Papers

**Malignant Neoplasms (Bone)**: Malignant neoplasms of bone which can occur in the craniomaxillofacial region almost always require complex surgical intervention. Many times bone and soft tissue are involved in these cases and the resections encompass a margin of uninvolved tissue to prevent recurrence. The difference between simple and complex may relate to the size of the area to be resected/reconstructed, the three-dimensionality of the affected area or its approximation to vital structures. Reconstruction for complex cases most times require a free flap and may at times also require patient-matched implants or patient-matched rigid fixation. ICD-10: C41.0 Malignant neoplasm of bones of skull and face, C41.1 Malignant neoplasm of mandible

PubMed Search: ((3D Printing) AND (Malignant Neoplasm Skull)) OR ((Rapid Prototyping) AND (Malignant Neoplasm Skull)) OR ((3D Printing) AND (Malignant Neoplasm Mandible)) OR ((Rapid Prototyping) AND (Malignant Neoplasm Mandible)) OR ((virtual surgical planning) AND (Malignant Neoplasm Mandible)) OR ((patient matched implant) AND (Malignant Neoplasm Mandible))

Results: [36, 55, 72, 73, 82, 88, 107, 111, 114, 161, 202–205, 246–300]

**Malignant Neoplasms (Soft Tissue)**: Malignant neoplasms of the soft tissue within the craniomaxillofacial region include cancers of the oral cavity (e.g. tongue, floor of mouth maxillary and mandibular gingiva), oropharynx, hypopharynx, orbit, skull base and larynx. Simple cases may only require biopsy while complex cases can include composite resection and reconstruction of soft tissue and hard tissue concomitantly. Reconstruction for complex cases may require autogenous tissue or a free flap and may at times also require patient-matched implants or rigid fixation. ICD-10: C00 Malignant Neoplasm of Lip, C01 Malignant Neoplasm of Base of Tongue, C04 Malignant Neoplasm of Floor of Mouth, C05 Malignant Neoplasm of Palate, C30 Malignant neoplasm of nasal cavity and middle ear, C31 Malignant neoplasm of accessory sinuses, C32 Malignant neoplasm of larynx, C33 Malignant neoplasm of trachea, D00.0 Carcinoma in situ of lip, oral cavity and pharynx, D00.1 Carcinoma in situ of esophagus

PubMed Search: ((3D Printing) AND (Malignant Neoplasm Lip)) OR ((Rapid Prototyping) AND (Malignant Neoplasm Lip)) OR ((3D Printing) AND (Malignant Neoplasm Tongue)) OR ((Rapid Prototyping) AND (Malignant Neoplasm Tongue)) OR ((3D Printing) AND (Malignant Neoplasm Palate)) OR ((Rapid Prototyping) AND (Malignant Neoplasm Palate)) OR ((3D Printing) AND (Malignant Neoplasm Sinus)) OR ((Rapid Prototyping) AND (Malignant Neoplasm Sinus)) OR ((3D Printing) AND (Malignant Neoplasm Larynx)) OR ((Rapid Prototyping) AND (Malignant Neoplasm Larynx)) OR ((3D Printing) AND (Malignant Neoplasm Trachea)) OR ((Rapid Prototyping) AND (Malignant Neoplasm Trachea)) OR ((3D Printing) AND (Carcinoma Lip)) OR ((Rapid Prototyping) AND (Carcinoma Lip)) OR ((3D Printing) AND (Carcinoma Pharynx)) OR ((Rapid Prototyping) AND (Carcinoma Pharynx)) OR ((3D Printing) AND (Carcinoma esophagus)) OR ((Rapid Prototyping) AND (Carcinoma Esophagus)) OR ((3D Printing) AND (Carcinoma Oral Cavity)) OR ((Rapid Prototyping) AND (Carcinoma Oral Cavity)) OR ((virtual surgical planning) AND (Malignant Neoplasm Base of Tongue)) OR ((patient matched implant) AND (Malignant Neoplasm Base of Tongue))

Results: [55, 150, 204, 247–250, 278, 279, 287, 290, 301, 302]

Genitourinary (Retrieved August 2017)

**Urolithiasis, Surgical or Medical Management**: Calculi or stones that form in the urinary tract, affecting the kidneys, ureters, bladder or urethra is common and increasing in prevalence due to a variety of proposed factors including obesity, dietary changes, and global warming. Terms associated with urolithiasis include kidney stones, renal stones, renal calculus disease, nephrolithiasis, calculi.

PubMed Search: (3D printing AND urolithiasis) OR (3D printing AND kidney stones) OR (3D printing AND renal stones) OR (3D printing AND renal calculus disease) OR (3D printing AND nephrolithiasis) OR (3D printing AND calculi) OR (rapid prototyping AND urolithiasis) OR (rapid prototyping AND kidney stones) OR (rapid prototyping AND renal stones) OR (rapid prototyping AND renal calculus disease) OR (rapid prototyping AND nephrolithiasis) OR (rapid prototyping AND calculi)

Results: [303–309]

**Renal Cancer**: Renal cancer is common, with renal cell carcinoma (RCC) accounting for approximately 3.5% of all malignancies [562]. In the US, there is predicted to be 63,990 new diagnoses of RCC and 14,400 kidney cancer related deaths in 2017. Surgical resection is the standard of care for RCC, with minimally invasive partial nephrectomy the treatment of choice for localized lesions [561].

PubMed Search: (3D printing AND kidney cancer) OR (rapid prototyping and kidney cancer) OR (3D printing AND renal cancer) OR (rapid prototyping AND renal cancer) OR (3D printing AND renal mass) OR (3D printing AND kidney mass) OR (rapid prototyping AND renal mass) OR (rapid prototyping AND kidney mass) OR (3D printing AND renal cell carcinoma) OR (3D printing AND RCC) OR (rapid prototyping AND renal cell carcinoma) OR (rapid prototyping AND RCC)

Results: [308, 310–324]

The terms renal lymphoma, angiomyolipoma (AML) and renal oncocytoma generated no results when searched with 3D printing or rapid prototyping.

**Renal Cysts**: Renal cysts are common; they are heterogeneous in both origin and pathogenesis; and most are simple and are usually of little clinical significance. Cystic renal disease may be sporadic, from congenital anomalies of the kidney and urinary tract that result in abnormal development of the renal parenchyma, or inherited, due to abnormal cilium signaling in tubular epithelial cells [561]. Inherited cystic renal diseases are now included in the group of diseases termed ciliopathies. Renal cysts are characterized based on the Bosniak classification system which divides cystic renal masses into five categories based on imaging characteristics. Simple cysts are considered Bosniak 1, minimally complex are Bosniak 2, intermediate are Bosniak 3, and malignant are Bosniak 4. Bosniak 3 and 4 lesions undergoing surgical treatment such as partial nephrectomy or radiofrequency ablation should be grouped in the renal cancer group described in the previous section.

PubMed Search: (3D printing AND Bosniak cystic lesions) or (rapid prototyping AND Bosniak cystic lesions) OR (3D printing AND Bosniak) OR (rapid prototyping AND Bosniak) OR (3D printing AND renal cysts) OR (rapid prototyping AND renal cysts) OR (3D printing AND kidney cyst) OR (rapid prototyping AND kidney cyst) OR (3D printing AND, cystic renal dysplasia) OR (rapid prototyping AND cystic renal dysplasia) OR (3D printing AND polycystic kidney disease) OR (rapid prototyping AND polycystic kidney disease)

Results: None

**Lower Tract Tumors (bladder and urethra) and Upper Tract Tumors (pyelocaliceal cavities and ureter)**: Urothelial carcinomas can be located in the lower (bladder and urethra) or the upper (pyelocalyceal cavities and ureter) urinary tract. Bladder cancer accounts for the majority of urothelial malignancies. In 2017, there are estimated to be 79,030 new cases of bladder cancer in the United States, 60,490 in men, making it the 4th most prevalent cancer in men [562]. Cancer of the ureter is uncommon and occurs most often in older adults who have been previously treated for bladder cancer. Transitional cell carcinoma is the most common histology observed.

PubMed Search: 3D printing AND urothelial malignancy OR rapid prototyping AND urothelial malignancy, 3D printing AND urothelial malignancies OR rapid prototyping AND urothelial malignancies, 3D printing AND urothelial carcinoma OR rapid prototyping AND urothelial carcinoma, 3D printing AND transitional cell carcinoma OR rapid prototyping AND transitional cell carcinoma, 3D printing AND bladder malignancy OR rapid prototyping AND bladder malignancy, 3D printing AND bladder malignancies OR rapid prototyping AND bladder malignancies, (3D printing AND bladder cancer OR rapid prototyping AND bladder cancer, 3D printing AND bladder neoplasm OR rapid prototyping AND bladder neoplasm, 3D printing AND bladder mass OR rapid prototyping AND bladder mass

Results: None Relevant

PubMed Search: (3D printing AND ureteral malignancy) OR (3D printing AND ureteral malignancies) OR (rapid prototyping AND ureteral malignancy) OR (rapid prototyping and ureteral malignancies) OR (3D printing AND intrarenal collecting system malignancies) OR (rapid prototyping AND intrarenal collecting system malignancies) OR (3D printing AND pyelocaliceal) OR (rapid prototyping AND pyelocaliceal) OR (3D printing AND pyelocalyceal) OR (rapid prototyping AND pyelocalyceal)

Results: None Relevant

**Adrenal Disease**: The adrenal glands may be affected by a variety of pathologies, the majority of which are benign. Causes of adrenal gland disorders include genetic mutations, tumors, infections, regulatory pathologies, or certain medications.

PubMed search: (3D printing AND adrenal disease) OR (rapid prototyping AND adrenal disease) OR (3D printing AND adrenal gland) OR (rapid prototyping AND adrenal gland)

Results: [325]

**Penile Cancer**: Cancer of the penis is an uncommon lesion occurring almost entirely in uncircumcised men. An important pathological process in penile cancer, is squamous cell carcinoma, which is caused by the human papillomavirus (HPV).

PubMed Search: (3D printing AND penile cancer) OR (rapid prototyping AND penile cancer)

Results: None

**Testicular Cancer**: The majorities (95%) of testicular tumors are derived from germ cells and all masses of the testes are considered malignant until proven otherwise [562].

PubMed Search: (3D printing AND testicular cancer) OR (rapid prototyping AND testicular cancer)

Results: None

**Prostate Cancer**: Prostate cancer is the most common cancer in American men, accounting for almost 1 in 5 new diagnoses [583]. Men diagnosed with prostate cancer have three primary treatment options including active surveillance, surgery, and radiation.

PubMed Search: (3D printing AND prostate cancer) OR (rapid prototyping AND prostate cancer)

Results: [326–334]

**Ovarian Disease**: Ovarian disease includes ovarian cancer and ovarian cysts, as well as polycystic ovarian syndrome.

PubMed Search: (3D printing AND ovarian tumor) OR (rapid prototyping AND ovarian tumor) OR (3D printing AND ovarian cancer) OR (rapid prototyping AND ovarian cancer)

Results: None Relevant

PubMed Search: (3D printing AND polycystic ovarian disease) OR (3D printing AND PCOD) OR (rapid prototyping AND polycystic ovarian disease) OR (rapid prototyping AND PCOD)

Results: None

**Uterine and Cervical Disease**: The uterine corpus is composed of endometrial mucosa and the underlying smooth muscle myometrium. Frequent and significant uterine disorders include endometriosis, adenomyosis, abnormal uterine bleeding, and lesions of the endometrium and myometrium including endometrial hyperplasia, endometrial carcinomas, endometrial polyps, and smooth muscle tumors.

PubMed Search: (3D printing AND uterine cancer) OR (rapid prototyping AND uterine cancer) OR (3D printing AND cervical cancer) OR (rapid prototyping AND cervical cancer)

Results: [335–342]

The terms endometrial adenocarcinoma, leiomyoma (uterine fibroids), leiomyosarcoma, and endometrial stromal sarcoma generated no results when searched with 3D printing or rapid prototyping.

PubMed Search: (3D printing AND endometriosis) OR (rapid prototyping AND endometriosis)

Results: [342]

PubMed Search: (3D printing AND endometritis) OR (3D printing AND adenomyosis) OR (3D printing AND uterine bleeding) OR (rapid prototyping AND endometritis) OR (rapid prototyping AND adenomyosis) OR (rapid prototyping) AND (uterine bleeding)

Results: None

**Vaginal Cancer**

PubMed Search: 3D printing AND vaginal tumor OR rapid prototyping AND vaginal tumor OR 3D printing AND vaginal cancer OR rapid prototyping AND vaginal cancer

Results: [339, 343]

**Genitourinary Reconstruction**: Genitourinary reconstruction encompasses a broad range of surgical procedures whose purpose is to correct congenital or acquired abnormalities. Terms: genitourinary conditions, genitourinary disorders, genitourinary anomalies, genitourinary abnormalities, genital conditions, genital disorders, genital anomalies, genital abnormalities.

PubMed Search: (3D printing AND genitourinary reconstruction) OR (rapid prototyping AND genitourinary reconstruction) OR (3D printing AND genitourinary disorders) OR (rapid prototyping AND genitourinary disorders) OR (3D printing AND genitourinary disorder) OR (rapid prototyping AND genitourinary disorder) OR (3D printing AND genitourinary anomaly) OR (rapid prototyping AND genitourinary anomaly) OR (3D printing AND genitourinary anomalies) OR (rapid prototyping AND genitourinary anomalies) OR (3D printing AND genitourinary abnormalities) OR (rapid prototyping AND genitourinary abnormalities) OR (3D printing AND genitourinary abnormality) OR (rapid prototyping AND genitourinary abnormality) OR (3D printing AND genital condition) OR (rapid prototyping AND genital condition) OR (3D printing AND genital anomaly) OR (rapid prototyping AND genital anomaly) OR (3D printing AND genital anomalies) OR (rapid prototyping AND genital anomalies) OR (3D printing AND genitourinary abnormalities) OR (rapid prototyping AND genitourinary abnormalities) OR (3D printing AND genitourinary abnormality) OR (rapid prototyping AND genitourinary abnormality)

Results: None Relevant

**Genitourinary Trauma**: Injury to the genitourinary tract is a common occurrence after both blunt and penetrating trauma. Terms: genitourinary injury, urinary tract trauma, renal trauma, kidney trauma (renal lacerations, renal collecting system injury, renal vascular injury), ureteral trauma, bladder trauma, urethral trauma, adrenal trauma, scrotal trauma.

PubMed Search: (3D printing AND genitourinary trauma) OR (rapid prototyping AND genitourinary trauma) OR (3D printing AND genitourinary injury) OR (additive manufacturing AND genitourinary injury) OR (3D printing AND urinary tract trauma) OR (additive manufacturing AND urinary tract trauma) OR (3D printing AND renal trauma) OR (rapid prototyping AND renal trauma) OR (3D printing AND kidney trauma) OR (rapid prototyping AND kidney trauma) OR (3D printing AND renal laceration) OR (rapid prototyping AND renal laceration) OR (3D printing AND renal collecting system injury) OR (rapid prototyping AND renal collecting system injury) OR (3D printing AND renal vascular injury) OR (rapid prototyping AND renal vascular injury) OR (3D printing AND ureteral trauma) OR (additive manufacturing AND ureteral trauma) OR (3D printing AND ureteral injury) OR (additive manufacturing AND ureteral injury) OR (3D printing AND bladder injury) OR (rapid prototyping AND bladder injury) OR (3D printing AND bladder trauma) OR (rapid prototyping AND bladder trauma) OR (3D printing AND adrenal trauma AND rapid prototyping AND adrenal trauma) OR (3d printing AND adrenal injury) OR (3D printing AND scrotal trauma) OR (rapid prototyping AND scrotal trauma) OR (3d printing AND scrotal injury)

Results: None Relevant

**Pediatric Infection and Reflux**: Acute pyelonephritis is inflammation of the kidney, usually bacterial in origin. Terms: vesicoureteral reflux (VUR), urinary tract infection (UTI), acute pyelonephritis

PubMed search: (3D printing AND vesicoureteral reflux) OR (rapid prototyping AND vesicoureteral reflux) OR (3D printing AND VUR) OR (rapid prototyping AND VUR)

Results: [344]

No results for PubMed search with 3D printing OR rapid prototyping AND the following: urinary tract infection, UTI, or pyelonephritis

**Pediatric Retroperitoneal Genitourinary Tumors**: The terms included Wilms tumor, nephroblastoma, and genitourinary tumor.

PubMed Search: (3D printing AND pediatric genitourinary tumor) OR (rapid prototyping AND pediatric genitourinary tumor) OR (3D printing AND Wilms tumor) OR (rapid prototyping AND Wilms tumor) OR (3D printing AND nephroblastoma) OR (rapid prototyping AND nephroblastoma)

Results: None Relevant

Musculoskeletal (Retrieved February 2017)

**Fracture**: Simple, Acute Complex Long Bone, Acute Complex Intraarticular, Complex, Acetabular, Non-Pathological Vertebral, Pathological Vertebral, Fracture Malunion.

PubMed Search: ((“printing, three-dimensional”[MeSH Terms] OR (“printing”[All Fields] AND “three-dimensional”[All Fields]) OR “three-dimensional printing”[All Fields] OR (“3d”[All Fields] AND “printing”[All Fields]) OR “3d printing”[All Fields]) OR (rapid[All Fields] AND prototyping[All Fields])) AND ((“fractures, bone”[MeSH Terms] OR (“fractures”[All Fields] AND “bone”[All Fields]) OR “bone fractures”[All Fields] OR “fracture”[All Fields]) OR malunion[All Fields])

Results: [345–406]

**Heterotopic Ossification**

PubMed Search: ((“printing, three-dimensional”[MeSH Terms] OR (“printing”[All Fields] AND “three-dimensional”[All Fields]) OR “three-dimensional printing”[All Fields] OR (“3d”[All Fields] AND “printing”[All Fields]) OR “3d printing”[All Fields]) OR (rapid[All Fields] AND prototyping[All Fields])) AND (“ossification, heterotopic”[MeSH Terms] OR (“ossification”[All Fields] AND “heterotopic”[All Fields]) OR “heterotopic ossification”[All Fields] OR (“heterotopic”[All Fields] AND “ossification”[All Fields]))

Results: None relevant

**Ligamentous Injury**

PubMed Search: ((“printing, three-dimensional”[MeSH Terms] OR (“printing”[All Fields] AND “three-dimensional”[All Fields]) OR “three-dimensional printing”[All Fields] OR (“3d”[All Fields] AND “printing”[All Fields]) OR “3d printing”[All Fields]) OR (rapid[All Fields] AND prototyping[All Fields])) AND ((“tendons”[MeSH Terms] OR “tendons”[All Fields] OR “tendon”[All Fields]) OR (“ligaments”[MeSH Terms] OR “ligaments”[All Fields] OR “ligament”[All Fields]))

Results: [407, 408]

**Hip Dysplasia**

PubMed Search: ((“printing, three-dimensional”[MeSH Terms] OR (“printing”[All Fields] AND “three-dimensional”[All Fields]) OR “three-dimensional printing”[All Fields] OR (“3d”[All Fields] AND “printing”[All Fields]) OR “3d printing”[All Fields]) OR (rapid[All Fields] AND prototyping[All Fields])) AND (“hip dislocation”[MeSH Terms] OR (“hip”[All Fields] AND “dislocation”[All Fields]) OR “hip dislocation”[All Fields] OR (“hip”[All Fields] AND “dysplasia”[All Fields]) OR “hip dysplasia”[All Fields])

Results: [389, 409–414]

**Bone or Soft Tissues Neoplasm: With or Without Joint and Neurovascular Involvement**

PubMed Search: ((“printing, three-dimensional”[MeSH Terms] OR (“printing”[All Fields] AND “three-dimensional”[All Fields]) OR “three-dimensional printing”[All Fields] OR (“3d”[All Fields] AND “printing”[All Fields]) OR “3d printing”[All Fields]) OR (rapid[All Fields] AND prototyping[All Fields])) AND (“bone neoplasms”[MeSH Terms] OR (“bone”[All Fields] AND “neoplasms”[All Fields]) OR “bone neoplasms”[All Fields] OR (“bone”[All Fields] AND “tumor”[All Fields]) OR “bone tumor”[All Fields])

Results: [324, 353, 415–443]

**Arthritis, Not Otherwise Specified**

PubMed Search: ((“printing, three-dimensional”[MeSH Terms] OR (“printing”[All Fields] AND “three-dimensional”[All Fields]) OR “three-dimensional printing”[All Fields] OR (“3d”[All Fields] AND “printing”[All Fields]) OR “3d printing”[All Fields]) OR (rapid[All Fields] AND prototyping[All Fields])) AND (“arthritis”[MeSH Terms] OR “arthritis”[All Fields])

Results: [358, 444–453]

**Scoliosis**: Secondary to Congenital Vertebral Anomaly, Severe/Marked, Thoracic Kyphosis, None of the Above

PubMed Search: ((“printing, three-dimensional”[MeSH Terms] OR (“printing”[All Fields] AND “three-dimensional”[All Fields]) OR “three-dimensional printing”[All Fields] OR (“3d”[All Fields] AND “printing”[All Fields]) OR “3d printing”[All Fields]) OR (rapid[All Fields] AND prototyping[All Fields])) AND (“scoliosis”[MeSH Terms] OR “scoliosis”[All Fields])

Results: [438, 454–463]

Vascular (Retrieved: Initial November 2017, Updated June 2018)

**Aortic Pathologies**: Dissection, Aneurysm, Stenting, Pseudoaneurysm

PubMed Search: ((“printing, three-dimensional”[MeSH Terms] OR (“printing”[All Fields] AND “three-dimensional”[All Fields]) OR “three-dimensional printing”[All Fields] OR (“3d”[All Fields] AND “printing”[All Fields]) OR “3d printing”[All Fields]) OR (rapid[All Fields] AND prototyping[All Fields])) AND ((“aneurysm, dissecting”[MeSH Terms] OR (“aneurysm”[All Fields] AND “dissecting”[All Fields]) OR “dissecting aneurysm”[All Fields] OR (“aortic”[All Fields] AND “dissection”[All Fields]) OR “aortic dissection”[All Fields]) OR (“aortic aneurysm”[MeSH Terms] OR (“aortic”[All Fields] AND “aneurysm”[All Fields]) OR “aortic aneurysm”[All Fields]) OR ((“aorta”[MeSH Terms] OR “aorta”[All Fields] OR “aortic”[All Fields]) AND (“stents”[MeSH Terms] OR “stents”[All Fields] OR “stent”[All Fields])) OR ((“aorta”[MeSH Terms] OR “aorta”[All Fields] OR “aortic”[All Fields]) AND (“aneurysm, false”[MeSH Terms] OR (“aneurysm”[All Fields] AND “false”[All Fields]) OR “false aneurysm”[All Fields] OR “pseudoaneurysm”[All Fields])))

Results: [464–499]

**Peripheral Aneurysm**

PubMed Search: ((“printing, three-dimensional”[MeSH Terms] OR (“printing”[All Fields] AND “three-dimensional”[All Fields]) OR “three-dimensional printing”[All Fields] OR (“3d”[All Fields] AND “printing”[All Fields]) OR “3d printing”[All Fields]) OR (Rapid[All Fields] AND Prototyping[All Fields])) AND ((“arteries”[MeSH Terms] OR “arteries”[All Fields] OR “arterial”[All Fields]) AND (“aneurysm”[MeSH Terms] OR “aneurysm”[All Fields]))

Results: [500–505]

**Stenosis, Arterial, Extracranial, for Patient-Specific Simulations**

PubMed Search: ((“printing, three-dimensional”[MeSH Terms] OR (“printing”[All Fields] AND “three-dimensional”[All Fields]) OR “three-dimensional printing”[All Fields] OR (“3d”[All Fields] AND “printing”[All Fields]) OR “3d printing”[All Fields]) OR (Rapid[All Fields] AND Prototyping[All Fields])) AND ((“constriction, pathologic”[MeSH Terms] OR (“constriction”[All Fields] AND “pathologic”[All Fields]) OR “pathologic constriction”[All Fields] OR “stenosis”[All Fields]) OR (“peripheral arterial disease”[MeSH Terms] OR (“peripheral”[All Fields] AND “arterial”[All Fields] AND “disease”[All Fields]) OR “peripheral arterial disease”[All Fields]))

Results: [506–508]

**Vascular Malformations**: Acquired, Congenital, Rings, Slings, For Interventional Consideration – Excluding congenital heart disease and intracranial pathologies.

PubMed Search: ((“printing, three-dimensional”[MeSH Terms] OR (“printing”[All Fields] AND “three-dimensional”[All Fields]) OR “three-dimensional printing”[All Fields] OR (“3d”[All Fields] AND “printing”[All Fields]) OR “3d printing”[All Fields]) OR (Rapid[All Fields] AND Prototyping[All Fields])) AND ((“vascular malformations”[MeSH Terms] OR (“vascular”[All Fields] AND “malformations”[All Fields]) OR “vascular malformations”[All Fields] OR (“vascular”[All Fields] AND “malformation”[All Fields]) OR “vascular malformation”[All Fields]) OR (“vascular ring”[MeSH Terms] OR (“vascular”[All Fields] AND “ring”[All Fields]) OR “vascular ring”[All Fields]) OR (“vascular ring”[MeSH Terms] OR (“vascular”[All Fields] AND “ring”[All Fields]) OR “vascular ring”[All Fields] OR (“vascular”[All Fields] AND “sling”[All Fields]) OR “vascular sling”[All Fields]))

Results: [509]

**Varices**: Peripheral for Medical Management, Retroperitoneal for Medical Management, Intervention Planning

PubMed Search: ((“printing, three-dimensional”[MeSH Terms] OR (“printing”[All Fields] AND “three-dimensional”[All Fields]) OR “three-dimensional printing”[All Fields] OR (“3d”[All Fields] AND “printing”[All Fields]) OR “3d printing”[All Fields]) OR (Rapid[All Fields] AND Prototyping[All Fields])) AND ((“varicose veins”[MeSH Terms] OR (“varicose”[All Fields] AND “veins”[All Fields]) OR “varicose veins”[All Fields] OR “varices”[All Fields]) OR (“varicose veins”[MeSH Terms] OR (“varicose”[All Fields] AND “veins”[All Fields]) OR “varicose veins”[All Fields] OR “varix”[All Fields]) OR varicose[All Fields])

Results: None

**Carotid Pathologies**: Stenosis, Dissection, Pseudoaneurysm, Post-Endarterectomy

PubMed Search: ((“printing, three-dimensional”[MeSH Terms] OR (“printing”[All Fields] AND “three-dimensional”[All Fields]) OR “three-dimensional printing”[All Fields] OR (“3d”[All Fields] AND “printing”[All Fields]) OR “3d printing”[All Fields]) OR (Rapid[All Fields] AND Prototyping[All Fields])) AND carotid[All Fields]

Results: [510–514]

**Intracranial Pathologies**: Stenosis, Aneurysm, Dural AV Fistula, Arteriovenous Malformation

PubMed Search: ((“printing, three-dimensional”[MeSH Terms] OR (“printing”[All Fields] AND “three-dimensional”[All Fields]) OR “three-dimensional printing”[All Fields] OR (“3d”[All Fields] AND “printing”[All Fields]) OR “3d printing”[All Fields]) OR (Rapid[All Fields] AND Prototyping[All Fields])) AND ((“constriction, pathologic”[MeSH Terms] OR (“constriction”[All Fields] AND “pathologic”[All Fields]) OR “pathologic constriction”[All Fields] OR “stenosis”[All Fields]) OR (“aneurysm”[MeSH Terms] OR “aneurysm”[All Fields]) OR (Dural[All Fields] AND (“arteriovenous fistula”[MeSH Terms] OR (“arteriovenous”[All Fields] AND “fistula”[All Fields]) OR “arteriovenous fistula”[All Fields] OR (“av”[All Fields] AND “fistula”[All Fields]) OR “av fistula”[All Fields])) OR (“arteriovenous malformations”[MeSH Terms] OR (“arteriovenous”[All Fields] AND “malformations”[All Fields]) OR “arteriovenous malformations”[All Fields] OR (“arteriovenous”[All Fields] AND “malformation”[All Fields]) OR “arteriovenous malformation”[All Fields]))

Results: [501, 515–545]

Breast (Retrieved November 2017)

**Benign breast lesions**: Benign breast lesions include fibrocystic change, benign breast masses, inflammatory, and peripartum conditions.

PubMed Search: ((3D printing) AND (fibrocystic change)) OR ((3D printing) AND (benign breast masses)) OR ((3D printing) AND (mastitis)) OR ((3D printing) AND (galactocele)) OR ((rapid prototyping) AND (fibrocystic change)) OR ((rapid prototyping) AND (benign breast masses)) OR ((rapid prototyping) AND (mastitis) OR ((rapid prototyping) AND (galactocele))

Results: None

**High risk breast lesions**: High risk lesions include flat epithelial atypia, atypical ductal hyperplasia, lobular neoplasia, radial scar, papillary lesions and mucocele-like lesions.

PubMed Search: ((3D printing) AND (flat epithelial atypia)) OR ((3D printing) AND (atypical ductal hyperplasia)) OR ((3D printing) AND (lobular neoplasia)) OR ((3D printing) AND (radial scar)) OR ((3D printing) AND (papillary lesions) OR ((3D printing) AND (mucocele-like lesions)) OR ((rapid prototyping) AND (flat epithelial atypia)) OR ((rapid prototyping) AND (atypical ductal hyperplasia)) OR ((rapid prototyping) AND (lobular neoplasia) OR ((rapid prototyping) AND (radial scar)) OR ((rapid prototyping) AND (papillary lesions)) OR ((rapid prototyping) AND (mucocele-like lesions))

Results: No results found.

**Breast cancer**: Malignant breast lesions included ductal carcinoma in situ ductal (DCIS) and invasive breast carcinomas. Use in breast malignancies with chest wall involvement and/or nipple-areolar complex involvement, evaluation of tumor-breast size ratio, and tumors where oncoplastic surgery is considered.

PubMed Search: ((3D printing) AND (breast cancer) OR ((rapid prototyping) AND (breast cancer))

Results: [546–552]

## Abbreviations

3D: Three-dimensional
ACR: American College of Radiology
ASD: Atrial septal defect
AV: Atrioventricular
CAD: Computer aided design
CDRH: The center for devices and radiological health
CHD: Congenital heart disease
CMF: Craniomaxillofacial
CNR: Contrast to noise ratio
CT: Computed tomography
DICOM: Digital imaging and communications in medicine
DOLV: Double-outlet left ventricle
DORV: Double-outlet right ventricle
EACTS-STS: European Association for Cardio-Thoracic Surgery / Society of Thoracic Surgery
FDA: The United States Food and Drug Administration
FOV: Field of view
HIPAA: Health Insurance Portability and Accountability Act
ICD-10: International Classification of Diseases, Tenth Revision
IPCCC: International Pediatric and Congenital Cardiac Code
MRI: Magnetic resonance imaging
NOS: Not otherwise specified
PACS: Picture archiving and communication system
PAPVR: Partial anomalous pulmonary venous return
ROI: Region of interest
RSNA: Radiological Society of North America
RVOT: Right ventricular outflow tract
SIG: Special Interest Group
SNR: Signal to noise ratio
STL: Standard tessellation language
TAPVR: Total anomalous pulmonary venous return
TGA: Transposition of the great arteries
VRML: Virtual reality markup language
VSD: Ventricular septal defect
